# Neuroligin-2 dependent conformational activation of collybistin reconstituted in supported hybrid membranes

**DOI:** 10.1074/jbc.RA120.015347

**Published:** 2021-01-13

**Authors:** Jonas Schäfer, Lucas Förster, Ingo Mey, Theofilos Papadopoulos, Nils Brose, Claudia Steinem

**Affiliations:** 1 Institute for Organic and Biomolecular Chemistry, Georg August University, Göttingen, Germany; 2 Department of Molecular Biology, University Medical Center, Göttingen, Germany; 3 Department of Molecular Neurobiology, Max Planck Institute of Experimental Medicine, Göttingen, Germany; 4 Max Planck Institute for Dynamics and Self-Organization, Göttingen, Germany

**Keywords:** GABAA receptor organization, inhibitory postsynapse, phosphoinositides, reflectometric interference spectroscopy (RIfS, ), supported hybrid membranes (SHMs), synapse, membrane, phosphatidylinositol, conformational change, GABA receptor

## Abstract

The assembly of the postsynaptic transmitter sensing machinery at inhibitory nerve cell synapses requires the intimate interplay between cell adhesion proteins, scaffold and adaptor proteins, and γ-aminobutyric acid (GABA) or glycine receptors. We developed an *in vitro* membrane system to reconstitute this process, to identify the essential protein components, and to define their mechanism of action, with a specific focus on the mechanism by which the cytosolic *C terminus* of the synaptic cell adhesion protein Neuroligin-2 alters the conformation of the adaptor protein Collybistin-2 and thereby controls Collybistin-2-interactions with phosphoinositides (PtdInsPs) in the plasma membrane. Supported hybrid membranes doped with different PtdInsPs and 1,2-dioleoyl-*sn*-glycero-3-{[*N*-(5-amino-1-carboxypentyl)iminodiacetic acid]succinyl} nickel salt (DGS-NTA(Ni)) to allow for the specific adsorption of the His_6_-tagged intracellular domain of Neuroligin-2 (His-*cyt*NL2) were prepared on hydrophobically functionalized silicon dioxide substrates via vesicle spreading. Two different collybistin variants, the WT protein (CB2_SH3_) and a mutant that adopts an intrinsically ‘open’ and activated conformation (CB2_SH3/W24A-E262A_), were bound to supported membranes in the absence or presence of His-*cyt*NL2. The corresponding binding data, obtained by reflectometric interference spectroscopy, show that the interaction of the *C terminus* of Neuroligin-2 with Collybistin-2 induces a conformational change in Collybistin-2 that promotes its interaction with distinct membrane PtdInsPs.

Synaptic signaling between neurons is based on the presynaptic release and postsynaptic sensing of neurotransmitters. In the mammalian brain, inhibitory synaptic signaling relies on the neurotransmitter γ-aminobutyric acid (GABA), which is detected by specific postsynaptic GABA_A_ receptors that operate as ligand-gated Cl^−^-channels. The clustering of these receptors in the postsynaptic plasma membrane, in direct apposition to the presynaptic transmitter release site, ensures fast signal transduction, so that GABA release induces a hyperpolarization of the postsynaptic membrane and reduced excitability (1, 2, 3, 4, 5, 6). Aberrant assembly and function of GABAergic synapses are the cause of multiple brain diseases (7, 8, 9, 10, 11, 12).

A defined protein machinery is required for GABA_A_ receptor clustering at many GABAergic postsynaptic sites (Fig. 1*A*). At the core of this machinery is the cell adhesion protein Neuroligin-2 (NL2) (13, 14), which interacts with presynaptic Neurexins (15, 16) and within postsynapses with the scaffolding protein Gephyrin (17, 18) and the adaptor protein Collybistin (CB). Previous studies showed that CB binds to phosphoinositides (PtdInsPs) via its pleckstrin homology (PH) domain (19, 20, 21, 22). On binding to PtdInsPs, CB serves as an adaptor to connect Gephyrin to the plasma membrane. This triggers Gephyrin oligomerization and the subsequent clustering of GABA_A_ receptors (14, 23, 24).

**Figure 1:**
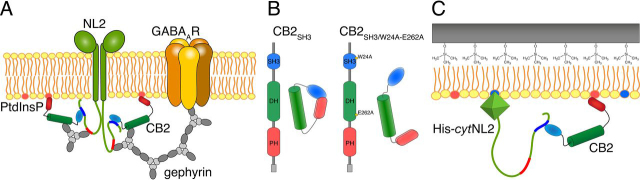
**The postulated NL2-CB2 interaction at inhibitory synapses and the design of the SHM assay.***A*, Schematic drawing illustrating the GABA_A_R clustering machinery at inhibitory postsynapses. *B*, Recombinant CB2_SH3_ proteins (WT CB2_SH3_ or the constitutively active CB2_SH3/W24A-E262A_ mutant) used in this study. *C*, Scheme of a supported hybrid membrane composed of an HMDS monolayer and a POPC monolayer (*yellow*) doped with DGS-NTA(Ni) (*blue*) and PtdInsPs (*red*) on a silicon dioxide substrate.

The adaptor protein CB, a guanine nucleotide exchange factor (GEF), is expressed in several splice variants that differ in their *N*- and *C termini* (CB1-CB3) and the presence or absence of a regulatory src homology 3 (SH3) domain (25). All CB variants contain a DH (Dbl homology) domain, which has GEF activity, and a *C*-terminal PH domain. Multiple studies demonstrated that mutations in CB cause neuronal dysfunction in various brain diseases. For example, an arginine to histidine exchange at position 290 within the DH domain affects the intramolecular interaction within the DH-PH tandem domain and thereby reduces the affinity of the PH domain to PtdIns[3]P, which is correlated to epileptic symptoms (21). Similarly, an arginine to tryptophan exchange at position 338 perturbs PH domain binding to PtdInsPs and causes a form of X-linked intellectual disability (26). These and other findings demonstrate that binding of the PH domain of CB to PtdInsPs is regulated by intramolecular interactions and of pivotal importance for the assembly of inhibitory postsynapses.

Previous studies (14) indicated that the most abundantly expressed, full-length, SH3-domain-containing CB isoform 2, CB2_SH3_, adopts a closed conformation (Fig. 1*B*), in which the SH3, DH, and PH domains interact intramolecularly and thus render the protein inactive, as is the case with the homologous GEFs Asaf1 and Asaf2 (27, 28). Disrupting this intramolecular interaction, *e.g.* by disabling the intramolecular binding sites in the CB2_SH3/W24A-E262A_ mutant (14), leads to a more open and active conformation, so that the PH domain can bind PtdInsPs. It has been hypothesized that in the biological context of the synapse the cytosolic NL2 *C terminus* binds the SH3 domain of CB2, thus activates it, and allows its interaction with plasma membrane PtdInsPs (29). However, this type of NL2-dependent CB2 activation has so far only been inferred from data obtained with corresponding knock-out neurons or with cultured neurons that express NL2 or CB2 variants that are either unable to interact or constitutively active. Clear molecular insights into the process have been lacking.

The present study was conducted to obtain direct molecular evidence of an NL2-mediated activation of CB2, leading to increased CB2 binding to plasma membrane PtdInsPs. To this end, we established an *in vitro* membrane system to reconstitute this putative key step in the development of inhibitory synapses. Specifically, we used supported hybrid membranes (SHMs) on hydrophobically functionalized silicon dioxide substrates via spreading of small unilamellar vesicles (SUVs). We showed recently that SHMs are superior to supported lipid bilayers as they provide a more homogeneous distribution of PtdInsPs (30). SHMs were doped with PtdInsPs serving as receptor lipids for CB2, whereas 1,2-dioleoyl-*sn*-glycero-3-{[*N*-(5-amino-1-carboxypentyl)iminodiacetic acid]succinyl} nickel salt (DGS-NTA(Ni)) was added to specifically adsorb the intracellular domain of NL2 (His-*cyt*NL2) via an *N*-terminally fused His_6_-tag (Fig. 1*C*). By means of reflectometric interference spectroscopy we were able to analyze the lipid-binding behavior of WT CB2_SH3_ and the intrinsically activated CB2_SH3/W24A-E262A_ mutant (14) in the absence or presence of His-*cyt*NL2. Our data show that the interaction of the *C terminus* of NL2 with CB2 induces a conformational change in CB2 that promotes its interaction with membrane PtdInsPs.

## Results

### Formation of supported hybrid membranes

In a first step, we produced lipid membranes on a silicon support to investigate and quantify the binding capability of CB2 to PtdInsPs in the presence or absence of the intracellular NL2 *C terminus*. We used a previously published protocol (30) to generate lipid monolayers composed of POPC and doped with different PtdInsPs. By using silicon dioxide substrates functionalized with 1,1,1-trimethyl-*N*-(TMS)silanamine (HMDS), to which small unilamellar vesicles (SUVs) were fused (Fig. 1*C*), a possible asymmetric distribution of PtdInsPs between the two lipid leaflets is prevented (30). Lipid monolayers composed of POPC and doped with 3 mol % of PtdIns[3]P, PtdIns[4,5]P_2_, or PtdIns[3,4,5]P_3_ on HMDS were prepared. The three PtdInsPs were chosen based on previous studies that had highlighted the involvement of these specific PtdInsPs as CB regulators in the formation of inhibitory synapses (19, 22, 31).

**Figure 2:**
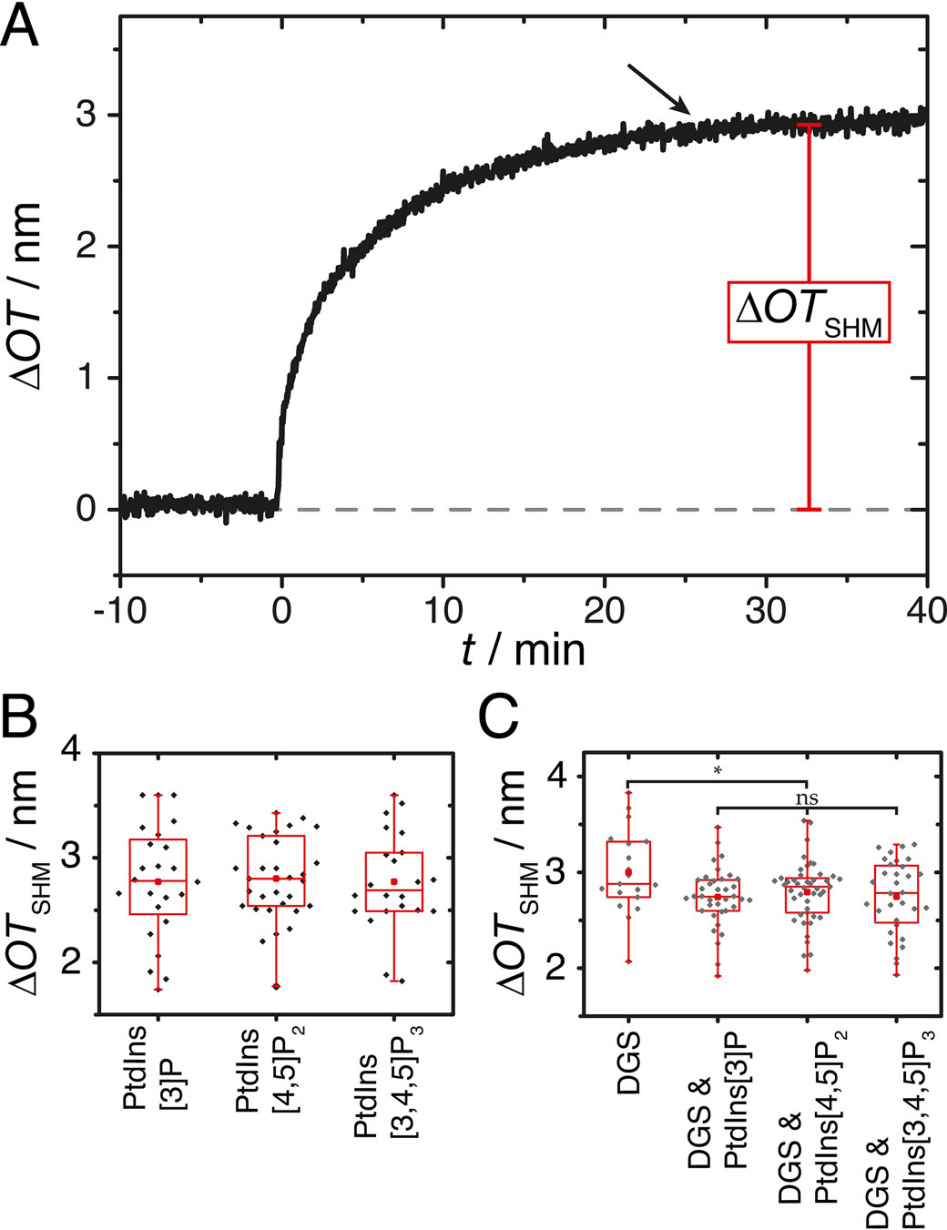
**Preparation of supported hybrid membranes on silicon dioxide surfaces with different PtdInsPs.***A*, Time resolved change in optical thickness Δ*OT*_SHM_ (marked in *red*) upon addition of SUVs composed of POPC/PtdIns[4,5]P_2_ (97:3, *n*/*n*) to an HMDS functionalized silicon dioxide surface at *t* = 0 min. The arrow indicates the time point of rinsing with buffer B. *B*, Box plots of Δ*OT*_SHM_ of POPC monolayers doped with 3 mol% of PtdInsP. *C*, Box plots of Δ*OT*_SHM_ of POPC monolayers doped with 3 mol% of PtdInsP and 3 mol% of DGS-NTA(Ni). The boxes extent from upper to lower quartile whereas the whiskers represent 1st and 99th percentiles. The medians are shown as horizontal lines and the means as red squares. DGS refers to DGS-NTA(Ni).

The spreading process of SUVs after HMDS functionalization was monitored in a time-resolved manner by reflectometric interference spectroscopy (RIfS). A characteristic time trace of the formation of a supported hybrid membrane (SHM) is depicted in Fig. 2*A*. Adsorption and spreading of the SUVs results in an increase in optical thickness (Δ*OT*) reaching a maximum at around 20 min. After monolayer formation, the system was rinsed with buffer B to remove excess lipid material and to adjust appropriate conditions for protein binding. Δ*OT*_SHM_ (Fig. 2*A*) is used as a quality parameter for the SHM preparation. For all three PtdInsPs doped POPC monolayers, Δ*OT*_SHM_ values of 2.77-2.80 nm were obtained, in good agreement with the expectation of a lipid monolayer on top of the HMDS monolayer. The mean values of Δ*OT*_SHM_ were (2.77 ± 0.11) nm for PtdIns[3]P (*n* = 24), (2.80 ± 0.07) nm for PtdIns[4,5]P_2_ (*n* = 31) and (2.77 ± 0.10) nm for PtdIns[3,4,5]P_3_ (*n* = 22), showing that the PtdInsP species does not influence the final monolayer thickness (Fig. 2*B*).

To follow the binding of the cytosolic domain of NL2 (His-*cyt*NL2) via its His_6_-tag to the membrane, we additionally prepared lipid monolayers composed of POPC, doped with 3 mol % of PtdInsP and 3 mol % of DGS-NTA(Ni). Again, Δ*OT*_SHM_ were readout from the RIfS experiments (Fig. 2*C*). Mean values of Δ*OT*_SHM_ were (2.74 ± 0.05) nm for DGS-NTA(Ni)/PtdIns[3]P (*n* = 38), (2.79 ± 0.05) nm for DGS-NTA(Ni)/PtdIns[4,5]P_2_ (*n* = 45) and (2.75 ± 0.07) nm for DGS-NTA(Ni)/PtdIns[3,4,5]P_3_ (*n* = 32). These results demonstrate that the addition of DGS-NTA(Ni) to the lipid composition does not alter the monolayer quality as deduced from the measured monolayer thickness. Only in case of the SHM composed of POPC and DGS-NTA(Ni), lacking a PtdInsP, a slightly larger mean Δ*OT*_SHM_ value (3.0 ± 0.1 nm, *n* = 19) was found.

**Figure 3:**
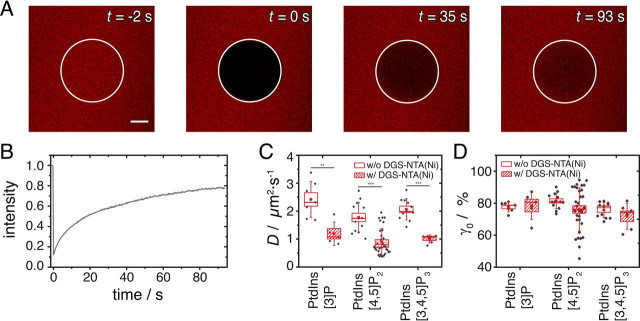
**FRAP experiment to access the mobility of PtdInsPs.***A,* Time lapse series of a FRAP experiment for an SHM composed of POPC/DGS-NTA(Ni)/PtdIns[4,5]P_2_/BODIPY®-TMR-PtdIns[4,5]P_2_, 94:3:2.7:0.3, *n/n*) at four different time points, with the bleached area indicated by a white circle. Scale bar: 5 μm. *B*, FRAP recovery curve for an SHM composed of POPC/DGS-NTA(Ni)/PtdIns[4,5]P_2_/BODIPY®-TMR-PtdIns[4,5]P_2_, 94:3:2.7:0.3, *n/n*). Box plots of the diffusion coefficients (*D*) in *C* and the mobile fractions (γ_0_) in *D* of the three different labeled PtdInsP in the presence or absence of DGS-NTA(Ni). The boxes represent the S.E. whereas the whiskers show the S.D. The medians are shown as horizontal lines and the means as red squares. Significant differences are indicated by ** *p* ≤ 0.01 and ***p ≤ 0.001.

We next tested whether the PtdInsP lipid mobility in the monolayer is influenced by the presence of the Ni^2+^-loaded DGS-NTA-lipid. Sufficient lipid mobility is a prerequisite to ensure that a lateral interaction between CB2 and NL2 at the membrane interface is possible. To investigate the lateral lipid mobility, we replaced 10% of the PtdInsPs by the corresponding BODIPY®-TMR labeled phosphoinositides and performed fluorescence recovery after photobleaching (FRAP) experiments. Fig. 3*A* shows a typical FRAP experiment of an SHM composed of POPC/DGS-NTA(Ni)/PtdIns[4,5]P_2_/BODIPY®-TMR-PtdIns[4,5]P_2_ (94:3:2.7:0.3, *n/n*). From the recovery curve (Fig. 3*B*), the diffusion coefficient *D* as well as the mobile fraction γ_0_ for each PtdInsP in the presence or absence of DGS-NTA(Ni) were calculated (Figs. 3C, D). Although the mobile fraction remains unaffected by the presence of DGS-NTA(Ni) (in the range of γ_0_ = 80%), the diffusion constants were decreased in the presence of DGS-NTA(Ni) by about 50%. For PtdIns[3]P, *D* is reduced from (2.4 ± 0.2) μm^2^s^−1^ (*n* = 7) without DGS-NTA(Ni) to (1.2 ± 0.2) μm^2^s^−1^ (*n* = 5) in its presence. A similar trend was detected for PtdIns[4,5]P_2_ and PtdIns[3,4,5]P_3_, with a decrease in *D* from (1.8 ± 0.2) μm^2^ s^−1^ (*n* = 13) to (0.9 ± 0.1) μm^2^ s^−1^ (*n* = 32) and from (2.1 ± 0.1) μm^2^ s^−1^ (*n* = 11) to (1.0 ± 0.1) μm^2^ s^−1^ (*n* = 6), respectively.

The reduced mobility of the PtdInsPs can be explained by a possible electrostatic interaction between the negatively charged PtdInsP and the DGS-NTA(Ni). However, clustering of the PtdInsP lipids, *i.e.* an inhomogeneity of the fluorescence intensity, was not resolved by confocal laser scanning microscopy (see Fig. 3*A* at *t* = -2 s). These results show that even in the presence of DGS-NTA(Ni) lateral mobility of the PtdInsPs is still ensured and lipid clusters of the size that could be resolved by fluorescence microscopy are not discernible. In conclusion, these data show that membranes containing both DGS-NTA(Ni) and the different PtdInsPs are suitable for the analyses of CB2 membrane binding and its dependence on His-*cyt*NL2.

### Isolation and purification of CB2 and NL2

**Figure 4:**
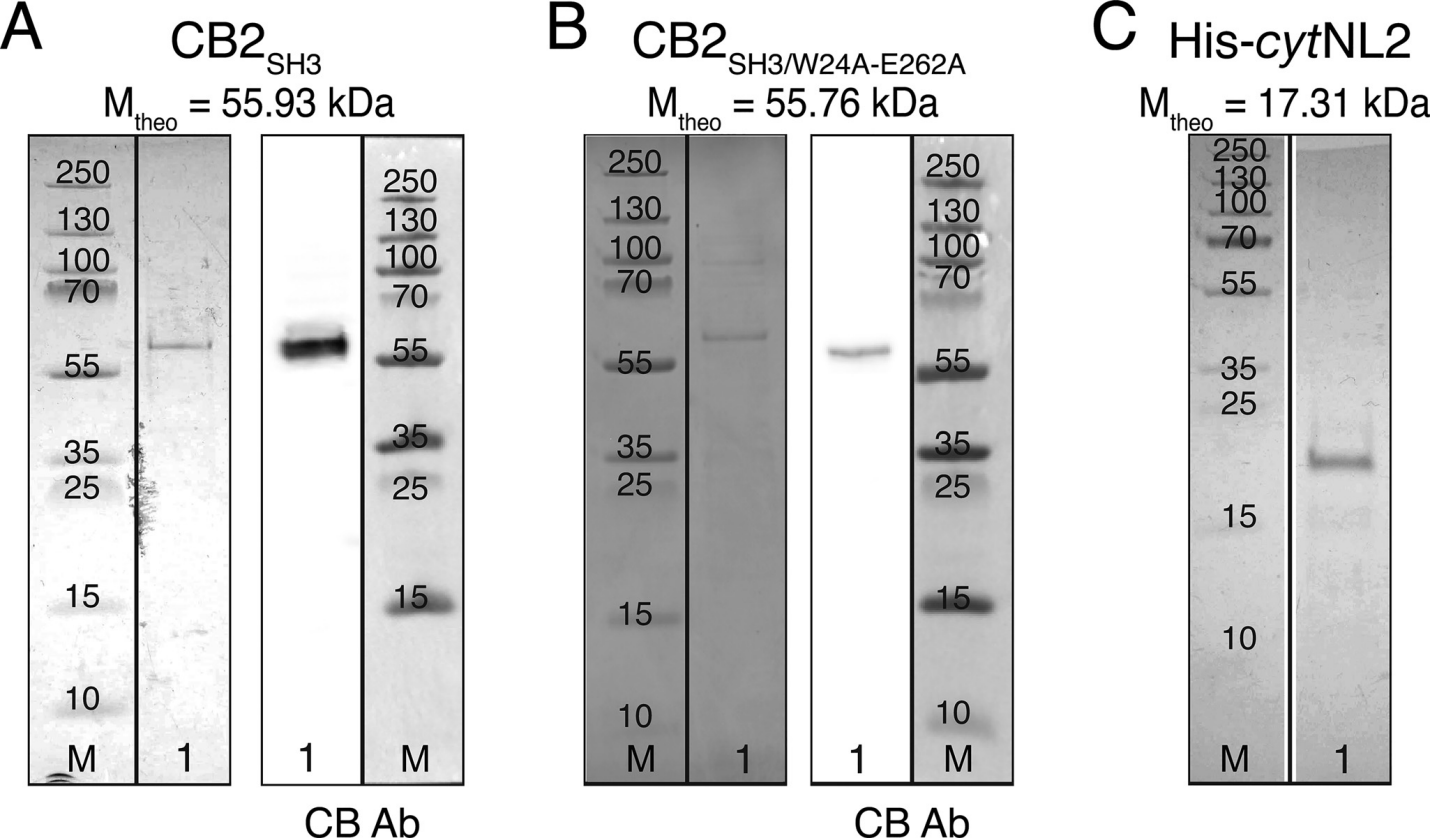
**Characterization of the different proteins used in this study.***A,* SDS-PAGE (*left*) and Western blotting overlay of chemiluminescence and marker image (*right*) of CB2_SH3_. *B*, SDS-PAGE (*left*) and Western blotting overlay of chemiluminescence and marker image (*right*) of CB2_SH3/W24A-E262A_. *C*, SDS-PAGE of His-*cyt*NL2. M: Marker, 1: protein sample. The vertical black lines mark the splice borders.

We next focused on the main aim of our study *i.e.* to assess using the established membrane system, whether the specific interaction of SH3-domain-containing CB2 (CB2_SH3_) with the cytosolic *C terminus* of NL2 (*cyt*NL2) leads to a conformational switch in CB2_SH3_ from a closed (autoinhibited) conformation to a more open (active) conformation that allows binding to PtdInsPs. To address this question, we recombinantly expressed and purified CB2_SH3_ and His-*cyt*NL2. As a positive control, we additionally purified the CB2_SH3/W24A-E262A_ mutant, which exhibits increased PtdInsP binding as compared with WT CB2_SH3_ (14, 32). Fig. 4 shows the results of SDS-polyacrylamide gel electrophoreses (SDS-PAGE) and Western blotting analyses (WB) of CB2_SH3_ (WT or the CB2_SH3/W24A-E262A_ mutant) and His-*cyt*NL2. After purification of both CB2 isoforms (via intein tags on a chitin resin column), a single protein band was identified in the SDS gels (Fig. 4*A*, *B*, left panel). A CB2 specific antibody (CB Ab, epitope: aa 44-229) identified these bands as CB2 (Fig. 4 *A*, *B*, right panel). For His-*cyt*NL2 purified on a Ni^2+^-nitrilotriacetic acid (NTA-(Ni^2+^)) agarose column and a subsequent anion exchange chromatography, a band at 17 kDa was detected in the SDS gels, in agreement with the theoretical mass of the protein (Fig. 4*C*).

### CB2 binding to phosphoinositides

**Figure 5:**
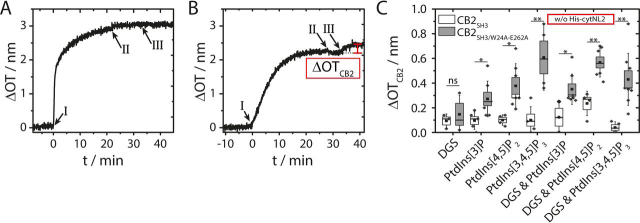
**Results of RIfS experiments to analyze the binding of CB2_SH3_ and CB2_SH3/W24A-E262A_ in the absence of *cyt*NL2.** Change in optical thickness (Δ*OT*) *versus* time during the adsorption of *A*, CB2_SH3_ and *B*, CB2_SH3/W24A-E262A_. The SHMs were composed of POPC/PtdIns[3,4,5]P_3_/DGS-NTA(Ni) (94:3:3, *n*/*n*). Monolayer formation was initiated by the addition of SUVs (I). After rinsing with buffer B (II), CB2 was added (III, 1 μm). The difference in OT (marked in *red*) is defined as Δ*OT*_CB2_. *C,* Box plots of Δ*OT*_CB2_ upon binding of CB2_SH3_ (white) and CB2_SH3/W24A-E262A_ (gray) to SHMs doped with 3 mol % of the different receptor lipids. The boxes represent the S.E. whereas the whiskers show the S.D. The medians are shown as horizontal lines and the means as central squares. Significant differences are indicated by * *p* ≤ 0.05 and **p ≤ 0.01. DGS is DGS-NTA(Ni).

**Figure 7:**
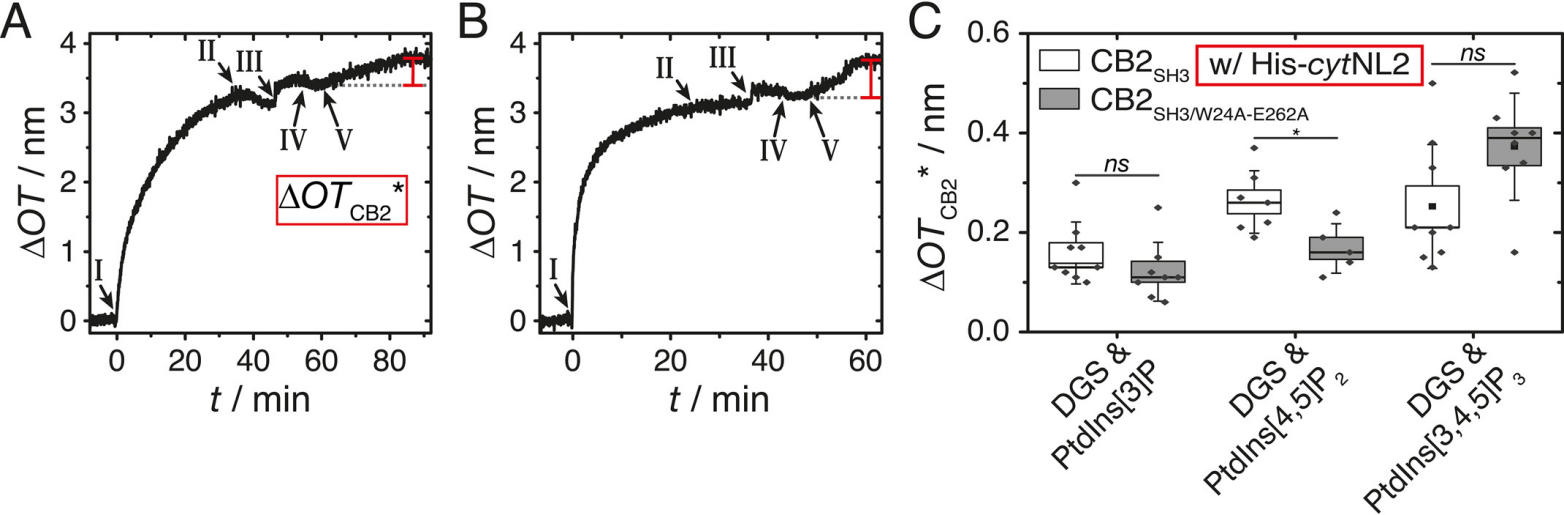
**Results of RIfS experiments to analyze the binding of CB2_SH3_ and CB2_SH3/W24A-E262A_ in the presence of *cyt*NL2.** Change in optical thickness (Δ*OT*) *versus* time during the adsorption of *A*, CB2_SH3_ and *B*, CB2_SH3/W24A-E262A_. The SHMs were composed of POPC/PtdIns[3,4,5]P_3_/DGS-NTA(Ni) (94:3:3, *n*/*n*). Monolayer formation was initiated by the addition of SUVs (I). After rinsing with buffer B (II), His-*cyt*NL2 was added (III, 1.36 μm). After a second rinsing with buffer B (IV), CB2 was added (V, 1 μm). The difference in OT (marked in *red*) is defined as Δ*OT*_CB2_*. *C,* Box plot of Δ*OT*_CB2_* upon binding of CB2_SH3_ (white) and CB2_SH3/W24A-E262A_ (gray) to SHMs doped with 3 mol % of the different receptor lipids after previous adsorption of His-*cyt*NL2. The boxes represent the S.E. whereas the whiskers show the S.D. The medians are shown as horizontal lines and the means as central squares. Significant differences are indicated by * *p* ≤ 0.05. DGS is DGS-NTA(Ni).

Based on the established SHMs, we investigated the binding of CB2 to the different PtdInsPs in the absence (Fig. 5) and in the presence (Fig. 7) of bound *cyt*NL2, whereas all other experimental conditions remain the same. Characteristic time traces of Δ*OT* upon SHM formation and CB2 addition (Δ*OT*_CB2_¸ definition see Fig. 5*B*) to a POPC/PtdIns[3,4,5]P_3_/DGS-NTA(Ni) (94:3:3, *n/n*) SHM in absence of *cyt*NL2 are shown in Fig. 5*A* (CB2_SH3_) and *B* (CB2_SH3/W24A-E262A_). A protein concentration of 1 μm was chosen for these experiments to obtain sufficiently high protein coverage without wasting too much protein (Fig. S1A, B). Different lipid compositions were used to elucidate the binding properties of WT CB2_SH3_ and the CB2_SH3/W24A-E262A_ mutant, which also allowed us to assess their nonspecific binding behavior. SHMs doped with only DGS-NTA(Ni) or with one of the three phosphoinositides were used or both receptor lipids were reconstituted simultaneously.

Δ*OT*_CB2_ for the different lipid compositions are depicted in Fig. 5*C* for CB2_SH3_ and CB2_SH3/W24A-E262A_. In case of CB2_SH3_, a small increase in Δ*OT* of about 0.1 nm was observed independently of the chosen lipid composition [DGS-NTA(Ni) or PtdInsP or a combination thereof] except for the POPC/PtdIns[4,5]P_2_/DGS-NTA(Ni) (94:3:3, *n*/*n*) membrane composition. The small Δ*OT*_CB2_ values of about 0.1 nm indicate that there is no specific interaction between CB2_SH3_ and the lipids analyzed. They can in part be ascribed to changes in the refractive index *n* caused by the addition of protein to the aqueous solution. On SHMs composed of POPC/PtdIns[4,5]P_2_/DGS-NTA(Ni) (94:3:3, *n*/*n*) a slightly larger Δ*OT*_CB2_ of (0.24 ± 0.05) nm (*n* = 4) was found indicating an increased amount of adsorbed CB2_SH3_. We can only speculate that the electrostatics on the surface is slightly different owing to an interaction of DGS-NTA(Ni) with PtdIns[4,5]P_2_. This can alter the position of the PtdIns[4,5]P headgroup protruding more from the membrane surface (33), and thus induces electrostatically driven interactions. For CB2_SH3/W24A-E262A_ significantly increased binding to the phosphoinositides was observed. Although there is a small nonspecific binding on DGS-NTA(Ni)-doped SHMs with Δ*OT*_CB2_ = (0.20 ± 0.09) nm (*n* = 3), CB2_SH3/W24A-E262A_ addition resulted in larger Δ*OT*_CB2_, well distinguishable from the base line level, when only phosphoinositides were present. For PtdIns[3]P and PtdIns[4,5]P_2_ containing SHMs, Δ*OT*_CB2_ was determined to be (0.28 ± 0.05) nm (*n* = 7) and (0.37 ± 0.07) nm (*n* = 7), respectively. The overall binding affinity given as a change in optical thickness at 1 μm protein concentration was largest for PtdIns[3,4,5]P_3_. Adsorption of CB2_SH3/W24A-E262A_ led to Δ*OT*_CB2_ of (0.67 ± 0.13) nm (*n* = 4). These results demonstrate that the CB2_SH3/W24A-E262A_ mutant can interact with the phosphoinositides presumably because of an open conformation induced by the mutations. In contrast, the WT CB2_SH3_ apparently remains in a closed, inactive conformation, rendering the protein incapable of interacting with PtdInsPs.

The adsorption of CB2_SH3/W24A-E262A_ in presence of DGS-NTA(Ni) resulted in Δ*OT*_CB2_ of (0.35 ± 0.04) nm (PtdIns[3]P, *n* = 8), (0.57 ± 0.05) nm (PtdIns[4,5]P_2_, *n* = 7) and (0.43 ± 0.07) nm (PtdIns[3,4,5]P_3_, *n* = 9) showing that DGS-NTA(Ni) does not significantly influence the binding of the active mutant.

### Binding of His-cytNL2 to the SHMs

**Figure 6:**
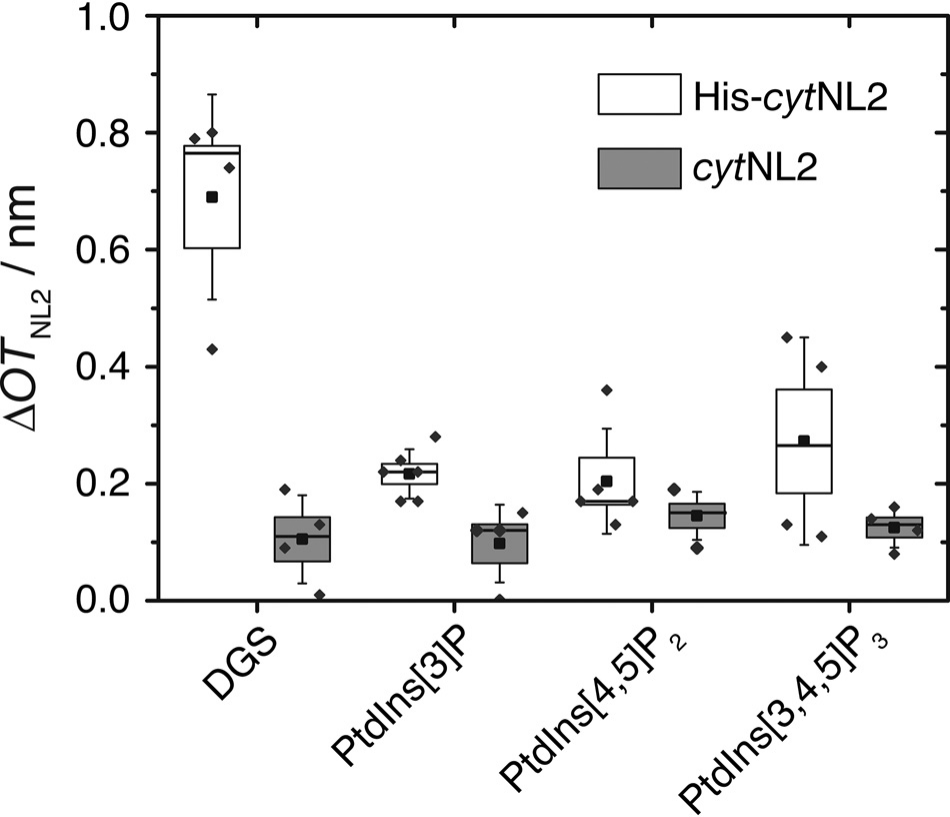
**Binding of *cyt*NL2 to different membrane compositions.** Box plot of the change in optical thickness (Δ*OT*_NL2_) upon addition of *cyt*NL2 (1.36 μm) with and without His_6_-tag (His-*cyt*NL2: white; *cyt*NL2: gray). The boxes represent the S.E. whereas the whiskers show the S.D. The medians are shown as horizontal lines and the means as central squares. DGS is DGS-NTA(Ni).

To induce the postulated conformational change in CB2_SH3_ upon interaction with the cytosolic part of NL2, the two proteins need to interact with each other at the membrane interface. Thus, we investigated whether His-*cyt*NL2 can be specifically bound via DGS-NTA(Ni) to the membrane. The specific adsorption of His-*cyt*NL2 to DGS-NTA(Ni) doped membranes was measured by RIfS (Fig. S1C). For a concentration of 1.36 μm a specific binding with a mean change in *OT* Δ*OT*_NL2_ = (0.69 ± 0.09) nm (*n* = 4) was observed (Fig. 6). To show the specificity of binding via the His_6_-tag to the DGS-NTA(Ni) lipid, the *N*-terminal His_6_-tag was cleaved off with the TEV (tobacco etch virus) protease (Fig. S2). After cleavage, only a small change of Δ*OT*_NL2_ = (0.10 ± 0.04) nm (*n* = 4) was observed.

We also investigated whether nonspecific binding of *cyt*NL2 occurs on phosphoinositide-doped membranes. Although *cyt*NL2 does not show any additional amount of binding, slightly larger Δ*OT*_NL2_ were observed for His-*cyt*NL2, which might be a result of the positively charged His_6_-tag interacting with the negatively charged PtdInsP lipids.

### Effect of membrane bound cytNL2 on CB2 activation

With the information that *cyt*NL2 is specifically bound to DGS-NTA(Ni), we now compare the binding of CB2_SH3_ to the different PtdInsPs in the absence (Fig. 5) with that in the presence (Fig. 7) of *cyt*NL2 under otherwise exact same conditions. The results allow us to address the question whether WT CB2_SH3_ is capable of binding to PtdInsP-doped membranes if *cyt*NL2 is present at the membrane interface. For a control, we also analyzed the binding behavior of the mutant CB2_SH3/W24A-E262A_, which is expected to bind to phosphoinositides even without any activation via *cyt*NL2. After His-*cyt*NL2 was bound to the SHMs doped with DGS-NTA(Ni) and one of the three PtdInsPs (III in Fig. 7*A* and *B*), either CB2_SH3_ or CB2_SH3/W24A-E262A_ was added (V in Fig. 7*A* and *B*), and the change in optical thickness (Δ*OT*_CB2_*, definition see Fig. 7*A*) was monitored by RIfS. In contrast to the result shown in Fig. 5*A*, where CB2_SH3_ was added to the same membrane but in the absence of *cyt*NL2, and where no change in OT was observed, a clear change in OT was found for CB2_SH3_ in the presence of *cyt*NL2 (Fig. 7*A*).

Fig. 7*C* summarizes the results for the three different PtdInsPs, which can be directly compared with the Δ*OT*_CB2_ values obtained in the absence of *cyt*NL2 (Fig. 5*C*, lanes PtdInsPs & DGS). The Δ*OT*_CB2_* values for CB2_SH3_ adsorption to PtdIns[3]P and PtdIns[4,5]P_2_ were determined to be (0.16 ± 0.02) nm (*n* = 9) and (0.26 ± 0.02) nm (*n* = 7), respectively. Compared with the Δ*OT*_CB2_ values (in absence of *cyt*NL2) for CB2_SH3_ adsorption to PtdIns[3]P ((0.09 ± 0.07) nm) and PtdIns[4,5]P_2_ ((0.24 ± 0.05) nm), there appears to be no significant difference in the amount of bound CB2_SH3_ if *cyt*NL2 is present. However, in case of PtdIns[3,4,5]P_3_ Δ*OT*_CB2_* was significantly (*p* < 0.01) increased to (0.25 ± 0.04) nm (*n* = 9) in presence of *cy*tNL2, as compared with Δ*OT*_CB2_ = (0.04 ± 0.02) nm (*n* = 4) in its absence. This result indicates an activation of the CB2_SH3_ WT rendering the protein capable of selectively interacting with PtdIns[3,4,5]P_3_.

However, there is another aspect that needs to be considered. A significantly (*p* < 0.001) reduced amount of the CB2_SH3/W24A-E262A_ mutant protein binds to the membrane surface in presence of *cyt*NL2 (Fig. 7*C*) compared with that found in its absence (Fig. 5*C*, lanes PtdInsPs & DGS). Δ*OT*_CB2_* was determined to be (0.12 ± 0.02) nm (*n* = 8) and (0.17 ± 0.02) nm (*n* = 5) for PtdIns[3]P and PtdIns[4,5]P_2_ containing SHMs, respectively (Fig. 7*C*). For comparison Δ*OT*_CB2_ = (0.35 ± 0.04) nm (PtdIns[3]P) and (0.57 ± 0.05) nm (PtdIns[4,5]P_2_) (Fig. 5*C*) was measured. Only in case of PtdIns[3,4,5]P_3_ doped membranes the overall amount of adsorbed CB2_SH3/W24A-E262A_ was not significantly influenced in the presence of *cyt*NL2 with Δ*OT*_CB2_* = (0.37 ± 0.04) nm (*n* = 8) compared with Δ*OT*_CB2_ = (0.43 ± 0.07) nm (*n* = 9) in its absence (compare Fig. 5*C* and Fig. 7*C*). These results indicate that the natively unfolded *cyt*NL2 occludes some of the PtdInsPs, possibly by ionic interactions. This inaccessibility of binding sites for CB2 leads to a reduction in binding capability of the CB2_SH3/W24A-E262A_ mutant. Taken the reduced Δ*OT*_CB2_* values into account, one can calculate the fraction of remaining available binding sites (ABS) in the presence of *cyt*NL2, defined as:

$$\text{ABS}={{\Delta \mathrm{OT}}_{\mathrm{CB2}}}^{*}\left(\mathrm{mutant}\right)/{\Delta \mathrm{OT}}_{\mathrm{CB2}}\left(\mathrm{mutant}\right),$$

resulting in 34% (PtdIns[3]P), 30% (PtdIns[4,5]P_2_) and 86% (PtdIns[3,4,5]P_3_). Thus, a direct comparison of CB2 WT adsorption in presence and absence of *cyt*NL2 appears not to be reasonable as *cyt*NL2's potential to compromise the CB2-PtdInsP interaction has to be considered. Despite this fact, the trend that the amount of bound CB2_SH3_ in the presence of *cyt*NL2 increases becomes obvious, if we compare the obtained Δ*OT*_CB2_ and Δ*OT*_CB2_* values of CB2_SH3_ related to the corresponding values for CB2_SH3/W24A-E262A_ that are measured under identical conditions and are set to 100%. This calculation leads to an increase of the relative amount of adsorbed CB2_SH3_ from 25% to 133% (PtdIns[3]P), from 42% to 153% (PtdIns[4,5]P_2_) and from 9% to 68% (PtdIns[3,4,5]P_3_).

## Discussion

The present study provides direct molecular evidence of an NL2-mediated activation of CB2, leading to increased CB2 binding to plasma membrane PtdInsPs.

Previous studies led to the hypothesis that the cytosolic NL2 *C terminus* binds the SH3 domain of CB2 in the biological context of the postsynapse, thereby inducing a more open conformation, which allows CB to interact with PtdInsPs located at the postsynaptic membrane (14, 29). By using SHMs, which provide a homogeneous distribution of PtdInsPs (30), we were able to establish an *in vitro* molecular model that allowed us to investigate directly how the interaction of CB2 with the cytosolic part of NL2 influences its ability to bind to different PtdInsPs. Our results show that, without the membrane-associated part of NL2, the WT protein CB2_SH3_ does not bind to any of the three PtdInsPs tested. This agrees with previous studies indicating that CB2_SH3_ adopts a closed, autoinhibited conformation, in which the PH domain is not accessible for PtdInsP binding (14, 32). As previously shown (14), the mutant protein CB2_SH3/W24A-E262A_, in which intramolecular interactions between the *C*-terminal PH and *N*-terminal SH3 domains of CB are weakened, adopts a more open conformation, which allowed the protein to interact with all three PtdInsPs used in the present study. We have chosen PtdIns[3]P as one of the receptor lipids based on previous studies highlighting the high affinity of CB2 to this PtdInsP (19, 32). The other two PtdInsPs are the most abundant ones in the post synaptic plasma membrane (34, 35) and present during postsynapse formation.

Whereas the interaction of CB with PtdIns[3]P allows the accumulation of CB and CB-associated proteins on early-endosomal membranes (22), the small GTPase TC10 binds to the PH domain of CB and induces a phospholipid affinity switch in CB, which allows CB to specifically interact with PtdInsP-species present at the plasma membrane, such as PtdIns[4,5]P_2_ and PtdIns[3,4,5]P_3_ (31, 36). Similarly to the interaction of NL2 with CB2_SH3_, the TC10-CB2_SH3_ interaction was previously suggested to interfere with intramolecular interactions between the different domains of CB2_SH3_, leading to a transition toward an open state of CB, which allows the PH domain to specifically bind to PtdInsPs located at the plasma membrane (36).

In order to induce the postulated conformational change in CB2_SH3_ upon interaction with the cytosolic part of NL2, the two proteins have to interact with each other at the membrane interface. To bind the cytosolic part of NL2 to the membrane, we exploited a His_6_-tag-DGS-NTA(Ni) strategy. Even though two oppositely charged lipids were inserted into the POPC matrix, a homogeneous SHM was produced, with laterally mobile lipids. However, the diffusion coefficients *D* of the BODIPY®-TMR PtdInsPs were reduced by about 50% compared with those without DGS-NTA(Ni). These diffusion coefficients are still in the range of those found in cellular plasma membranes of fibroblasts and epithelial cells with an average of *D* = (0.8 ± 0.2) μm^2^/s (37) and hence appear to be sufficient to allow for lateral protein-protein interactions at the membrane interface.

Our comparative analysis of the binding of CB2_SH3_ (WT and the W24A-E262A mutant) to PtdInsPs in the presence or absence of *cyt*NL2 clearly indicates that the cytosolic *C terminus* of NL2 alters the conformation of CB2 and thereby controls CB2-interactions with PtdInsPs at the plasma membrane. In the absence of *cyt*NL2, only the “open conformation-mutant”, CB2_SH3/W24A-E262A_, efficiently interacted with PtdInsPs. In contrast, in the presence of *cyt*NL2, similar amounts of WT CB2_SH3_ and the CB2_SH3/W24A-E262A_ mutant bound to PtdInsPs. However, there is an overall decrease in the amount of CB2_SH3/W24A-E262A_ bound to the three PtdInsPs in the presence of *cyt*NL2, as compared with that in the absence of membrane anchored *cyt*NL2. One cannot rule out that the W24A-E262A double-mutation of CB_SH3_ alters the specificity for the different PtdInsPs compared with the WT protein, as shown previously for CB and other proteins (38, 39). Moreover, in agreement with this hypothesis, a previous study indicated that a single (R290H) mutation in the DH domain of CB, which leads to epilepsy and intellectual disability in humans, alters the strength of intramolecular interactions between the DH and the PH domains of CB, thereby leading to a reduced PtdIns[3]P-binding of CB (21). Thus, whereas the interaction of WT CB_SH3_ with endogenous activator-proteins, such as the cell-adhesion protein NL2 studied here or the small Rho-like GTPase TC10 (31), leads to a fine-tuned increase of the interaction of CB2 with certain PtdInsPs enriched at the plasma membrane, the “open-conformation mutant” CB2_SH3/W24A-E262A_ might lead to a more general and less specific increase of CB binding to a broader range of PtdInsPs.

As regards WT CB2_SH3_, an interesting finding of our study is that for PtdIns[3,4,5]P_3_ the Δ*OT*_CB2_* (in the presence of *cyt*NL2) was significantly increased, as compared with the Δ*OT*_CB2_ (in the absence of *cyt*NL2). For PtdIns[3]P and PtdIns[4,5]P_2_, a similar trend toward increased Δ*OT*_CB2_* was observed but did not reach significance, as compared with Δ*OT*_CB2_. However, for both, WT CB2_SH3_ and the W24A-E262A mutant, it appears that the PtdInsP-accessibility is reduced upon His-*cyt*NL2 binding to the membrane, resulting in an overall decrease in the amount of bound CB2 proteins. If this is assumed, we can relate the Δ*OT*_CB2_ values obtained in the absence of *cyt*NL2 to the changes in optical thickness for CB2_SH3_ in presence of *cyt*NL2 and calculate an increase in the amount of bound CB2_SH3_ for all three PtdInsPs with a maximum increase for PtdIns[3,4,5]P_3_. This agrees with a previous study, indicating that endogenous CB2 activators can induce a switch in the conformation of CB2, which allows enhanced interaction with plasma membrane-PtdInsPs (31). Thus, we conclude that in the presence of the cytosolic domain of NL2 a switch from a closed to an open conformation of CB2 is induced, which enables CB2 to properly anchor at nascent inhibitory postsynapses enriched in PtdIns[4,5]P_2_ and PtdIns[3,4,5]P_3_. It is likely that this conformational switch is induced by the interaction of the *N*-terminal SH3 domain of CB with poly-proline sequences in *cyt*NL2 (13, 40). As we only adsorbed the water-soluble cytosolic domain of NL2, we moreover conclude that a dimerization of NL2 via the transmembrane domains, as it was observed for NL2 *in vivo* (41), is not required for CB2 activation.

## Experimental Procedures

### Materials

C_16_ derivatives and BODIPY®-TMR labeled derivatives of PtdInsPs (PtdIns[3]P, PtdIns[4,5]P_2_, and PtdIns[3,4,5]P_3_) were obtained as from Echelon Biosciences (Salt Lake City, UT). 1-palmitoyl-2-oleoyl-*sn*-glycero-phosphocholine (POPC) and 1,2-dioleoyl-*sn*-glycero-3-{[*N*-(5-amino-1-carboxypentyl)iminodiacetic acid]succinyl} nickel salt (DGS-NTA(Ni)) were purchased from Avanti Polar Lipids (Alabaster, Alabama). BL21(DE3) competent *Escherichia coli* cells were purchased from Invitrogen whereas BL21(DE3) Rosetta competent *E. coli* cells were from VWR International (Darmstadt, Germany). Chitin resin was obtained from New England Biolabs (Ipswich, MA) and NTA(Ni^2+^) agarose (Protino™) from Macherey-Nagel (Düren, Germany). Antibodies specific for CB2 (1: 1000, Cat. No. 261 011) and NL2 (1: 1000, Cat. No. 129 213) were purchased from Synaptic Systems (Göttingen, Germany) whereas the His-tag antibody (1: 1000, ab18184) was obtained from Abcam (Cambridge, UK). Silicon wafers were purchased from Silicon Materials (Kaufering, Germany). 1,1,1-Trimethyl-*N*-(TMS)silanamine (HMDS) was purchased from VWR International (Darmstadt, Germany).

### Protein purification

Proteins were recombinantly expressed in *E. coli* following previously described protocols (14, 42). Briefly, His-*cyt*NL2 was obtained from transformed *E. coli* BL21(DE3) Rosetta cells containing the bacterial expression vector pETM11 (EMBL, Heidelberg, Germany). The plasmid was kindly provided by the group of Hermann Schindelin (Rudolf-Virchow-Zentrum, Würzburg, Germany). It encodes the intracellular domain of NL2 with an *N*-terminally fused histidine tag. First, the cells were grown to an OD_600_ = 0.8 in kanamycin (50 μg·ml^−1^) containing LB medium. Protein expression was induced by addition of 0.5 mm isopropyl-β-d-thiogalactopyranoside (IPTG). After incubation for ≥15 h at 15 °C, the cells were harvested by centrifugation (4,000 × *g*, 20 min, 4 °C), and the pellet was resuspended in lysis buffer A (500 mm NaCl, 100 mm HEPES, 10%(*v/v*) glycerol, 6 mm benzamidine, 5 mm mercaptoethanol, 2 mm PMSF, protease inhibitor mixture (cOmplete; Roche Diagnostics, Basel, Switzerland, pH 8.0). Lysis was completed using a microfluidizer (1 kbar, three cycles, ice cooled, LM10 processor, Microfluidics, Westwood, MA). Afterward, the bacterial lysate was clarified by centrifugation (57,500 × *g*, 45 min, 4 °C). The supernatant was then applied to a Ni^2+^-nitrilotriacetic acid (NTA-(Ni^2+^)) agarose column and incubated for 30 min. Then the column was washed with 20 CV lysis buffer A and wash buffer A (500 mm NaCl, 100 mm HEPES, 10%(*w/v*) glycerol, 5 mm mercaptoethanol, pH 8.0) each. His-*cyt*NL2 was eluted using buffer A (250 mm NaCl, 250 mm imidazole, 500 mm HEPES, 5 mm mercaptoethanol, pH 8.0) after incubation for 30 min. The protein solution was then dialyzed against imidazole-free Äkta-buffer (50 mm NaCl, 25 mm HEPES, 1 mm EDTA, pH 8.0) and applied to an anion-exchange chromatography column (Mono Q 5/50 GL, GE Healthcare, Uppsala, Sweden). The protein was eluted with an ion gradient created by use of Äkta-buffer containing 2 m NaCl. The purified protein was dialyzed against buffer B (100 mm NaCl, 25 mm HEPES, pH 8.0) and stored at 4 °C until use after its concentration was determined via Bradford test.

CB2_SH3_ and CB2_SH3/W24A-E262A_ were obtained from transformed *E. coli* BL21(DE3) cells. Plasmids based on the IMPACT system vector pTXB1 encoding both full-length forms of CB2 with *C*-terminal intein tags were transformed into *E. coli* cells and cells were grown to an OD_600_ of 0.9–1.0 at 37 °C in LB-Miller medium containing ampicillin (100 μg·ml^−1^). Protein expression was induced by addition of 0.5 mm IPTG. After overnight incubation at 15 °C, cells were harvested by centrifugation (4,000 × *g*, 20 min, 4 °C). The cell pellets were resuspended in lysis buffer B (250 mm NaCl, 20 mm HEPES, 2 mm EDTA, 10%(*w*/*v*) glycerol, pH 8.0), and cell lysis was performed using a microfluidizer (1 kbar, three cycles, ice cooled; LM10 processor, Microfluidics, Westwood, MA, USA). After centrifugation (70,000 × *g*, 30 min, 4 °C), the supernatant was applied to the equilibrated chitin resin column for 1 h. The resin was rinsed with 500 ml of wash buffer B (1 m NaCl, 20 mm HEPES, 2 mm EDTA, pH 8.0), and protein cleavage was induced by incubation with 50 mm DTT in buffer C (250 mm NaCl, 20 mm HEPES, 2 mm EDTA, pH 8.0) for > 24 h. Finally, the protein was eluted with buffer C containing 5 mm DTT. Concentration and dialysis to buffer B (100 mm NaCl, 25 mm HEPES, pH 8.0) was performed by ultrafiltration using spin concentrators (Sartorius, Göttingen, Germany). Protein concentrations were determined by UV/Vis spectroscopy using extinction coefficients of ε_280_(CB2_SH3_) = 98,945 m^−1^cm^−1^ and ε_280_(CB2_SH3/W24A-E262A_) = 93,445 m^−1^cm^−1^.

All proteins were analyzed by SDS-PAGE and Western Blots using a Gel-Imager (Azure c300, azure biosystems, Dublin, USA) for documentation.

### Substrate preparation

Silicon substrates with a SiO_2_ layer thickness of 5 μm were cleaned two times with detergent solution followed by rinsing with ultrapure water in an ultrasonic bath for 15 min each. Afterward, the substrates were treated with O_2_-plasma for 30 s and then exposed to hexamethyldisilazane (HMDS) as previously described in detail (30).

### Vesicle preparation

A stock solution of POPC was prepared in chloroform at a concentration of 10 mg·ml^−1^. Lyophilized PtdInsPs were dissolved in mixtures of chloroform/methanol/water to concentrations of 1 mg·ml^−1^. Lipid stock solutions (0.4–0.8 mg of total lipid material) were mixed in a test tube preloaded with 100 μl chloroform at the desired molar ratio. Fluorophores were added as indicated. Organic solvent was evaporated with a gentle stream of nitrogen at 25 °C. To remove residual solvent, the lipid film was dried under vacuum for 3 h at the corresponding temperature. Lipid films were stored at 4 °C until use. A lipid film was rehydrated by adding 0.5–1.2 ml of spreading buffer (20 mm citrate, 50 mm KCl, 0.1 mm NaN_3_, pH 4.8) and incubated for 30 min. Multilamellar vesicles (MLVs) were obtained by vortexing for 3 × 30 s at 5 min intervals. The MLV suspension was transferred to an Eppendorf cup and sonicated for 30 min using an ultrasonic homogenizer (Sonopuls HD2070, resonator cup; Bandelin, Berlin, Germany) to obtain small unilamellar vesicles (SUVs).

### Reflectometric interference spectroscopy (RIfS)

RIfS is a label-free, noninvasive technique determining the optical thickness (*n* × *d*) of a thin layer by measuring white light interference. This interference is caused by partial reflection at interfaces whose distance is within the coherence length of white light (43). RIfS was employed to monitor the formation of SHMs and subsequent protein adsorption to receptor lipid containing membranes in a label-free and time-resolved manner. The experimental setup is described in detail elsewhere (44). Briefly, a Flame-S-UV/Vis spectrometer (Ocean Optics, Dunedin, FL, USA) was used to record interference spectra at intervals of 2 s. Data were evaluated applying a MATLAB (The MathWorks, Natick, MA, USA) tool following the work of Krick *et al.* (44).

### Confocal laser scanning microscopy (CLSM)

CLSM images were taken with a confocal laser scanning microscope LSM 880 (Carl Zeiss Microscopy GmbH, Oberkochen, Germany) equipped with a 40× objective (W Plan-Apochromat, NA = 1.0, Zeiss). BODIPY®-TMR-PtdInsPs were monitored at 520 − 650 nm after excitation at 488 nm.

### Fluorescence recovery after photobleaching (FRAP)

Fluorescence intensity in a region of interest (ROI) of a model membrane doped with one of the BODIPY®-TMR-PtdInsPs (λ_bleach_ = 488 nm) was bleached by a short laser pulse, and the time-dependent fluorescence recovery was recorded with a frame rate of 3-4 frames·s^−1^. The diffusion coefficients and mobile fractions were calculated using a Hankel transformation (45).

## Data availability

All data are contained within the article.
