# Supplementary material for: Neuroligin-2 dependent conformational activation of collybistin reconstituted in supported hybrid membranes
Source: J Biol Chem. 2021 Jan 13;295(52):18604–13. doi: 10.1074/jbc.RA120.015347 (PMC7939476; doi:10.1074/jbc.RA120.015347)
Supplement: Supplementary file 1 [file mmc1.pdf]

Supporting Information

Neuroligin-2 dependent conformational activation of Collybistin reconstituted in supported hybrid membranes

Jonas Schäfer[1], Lucas Förster[1], Ingo Mey[1], Theofilos Papadopoulos[2], Nils Brose[3*], Claudia Steinem[1,4*]

[1] Institute for Organic and Biomolecular Chemistry, Georg August University, Göttingen, Germany

[2] Department of Molecular Biology, University Medical Center, Göttingen, Germany

[3] Department of Molecular Neurobiology, Max Planck Institute of Experimental Medicine, Göttingen, Germany

[4] Max Planck Institute for Dynamics and Self-Organization, Göttingen, Germany

Adsorption isotherms

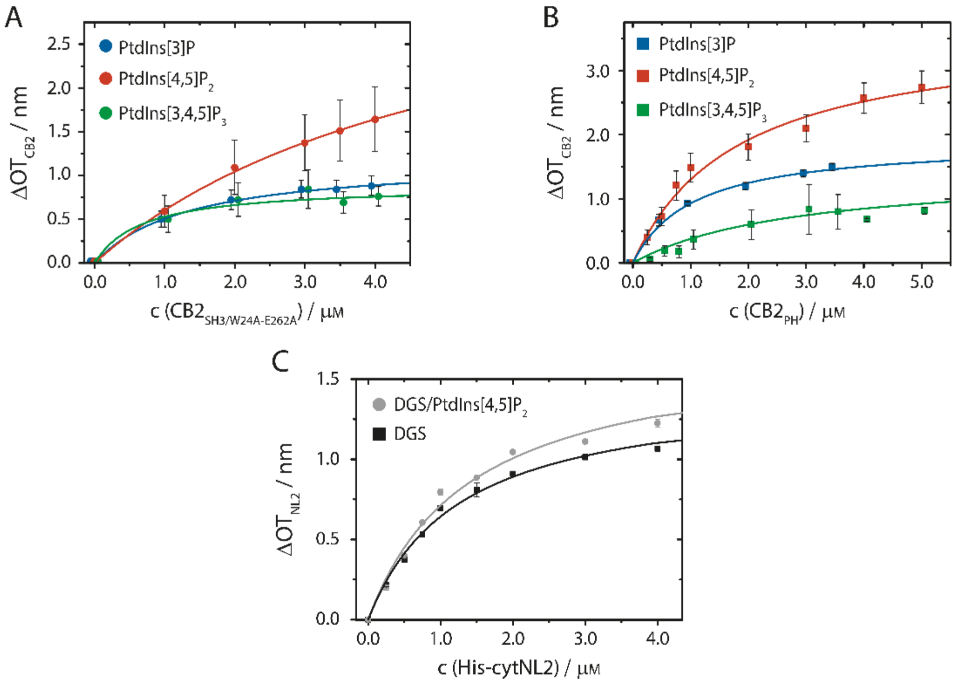

FigureS1. Adsorption isotherms of $CB2_{SH3/W24A-E262A}$ (*A*), $CB2_{PH}$ (*B*) and His-*cyt*NL2 (C) on POPC SHMs doped with different receptor lipids. SHMs composed of POPC and doped with 3 mol% of the lipid as indicated in the legends were used. Adsorption isotherms were obtained by stepwise increase in protein concentration.

We performed concentration dependent experiments with $CB2_{SH3/W24A-E262A}$ (Fig. S1*A*) for all three PtdInsPs under investigation. From the obtained adsorption isotherms of $CB2_{SH3/W24A-E262A}$, we concluded that a protein concentration of 1 µM is sufficient to obtain high protein coverage without wasting too much protein. We also recorded adsorption isotherms of the PH domain of CB2 ($CB2_{PH}$) for all three PtdInsPs (Fig. S1*B*) to show the performance of the RIfS technique.

Adsorption isotherms for His-*cyt*NL2 were measured in presence and absence of PtdIns[4,5]P_2 (Fig. S1*C*). Based on the obtained isotherms, we first used a His-*cyt*NL2 concentration of 4 µM. However, it turned out that binding of His-*cyt*NL2 partially blocks the interaction of $CB2_{SH3/W24A\text{-}E262A}$ with the PtdInsPs. Hence, we reduced the His-*cyt*NL2 concentration to 1.36 µM, which still leads to a significant amount of bound protein according to the adsorption isotherms but reduces the *cyt*NL2 surface coverage, which reduces also part of the blocking of the PtdInsP binding sites.

Western blot analysis of TEV cleavage

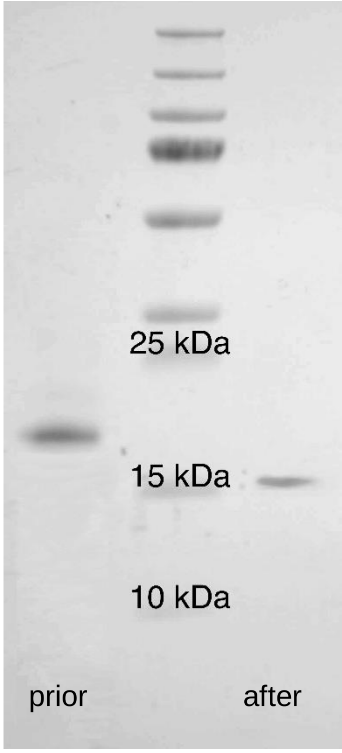

Figure S2. Western blot of *cyt*NL2 prior and after TEV cleavage.
